# Biodegradable polymeric microsphere formulations of full-length anti-VEGF antibody bevacizumab for sustained intraocular delivery

**DOI:** 10.1007/s13346-025-01795-y

**Published:** 2025-01-24

**Authors:** Shwetha Iyer, Cameron Lee, Mansoor M. Amiji

**Affiliations:** 1 Department of Pharmaceutical Sciences, School of Pharmacy and Pharmaceutical Sciences, Northeastern University, Boston, MA 02115 USA; 2 Novartis Biomedical Research, Cambridge, MA 02139 USA; 3 Department of Chemical Engineering, College of Engineering, Northeastern University, Boston, MA 02115 USA

**Keywords:** Anti-VEGF antibody, Bevacizumab, Intraocular delivery, Polymeric microspheres, Sustained release, Age-related macular degeneration

## Abstract

Age-related macular degeneration (AMD) is one of the leading causes of central vision loss in the elderly population. Bevacizumab, a full-length humanized monoclonal anti-VEGF antibody, is commonly used off-label drug to treat AMD. However, the dosing regimen of bevacizumab and other anti-VEGF antibodies requires monthly intravitreal injections followed by regular intravitreal injections at 4–16-week intervals. In 2021, the FDA approved an innovative port delivery system of ranibizumab (Susvimo^®^) that can be implanted intravitreally to slowly release the active ingredient anti-VEGF antibody and reduce injection frequency to once every 6 months. An approach utilizing polymeric slow-release microspheres encapsulating a full-length antibody, such as bevacizumab, would be much more patient-friendly because it could be injected intravitreally, avoiding surgical implantation. While microsphere encapsulation is traditionally successful for small molecule hydrophobic drugs, we assessed two different polymers, namely poly(D, L-lactide-co-glycolide) (PLGA) and poly(epsilon-caprolactone) (PCL) and discovered the benefits of utilizing a slow degrading hydrophobic polymer such as PCL for large protein therapeutic. Using the traditional double emulsion fabrication method with PCL polymer, we could produce microspheres that encapsulate bevacizumab antibody and demonstrate the release of biologically active therapeutic agent for up to 60 days. This novel approach could lead to significant advancements in our field and potentially open new avenues for future research.

**Graphical Abstract:**

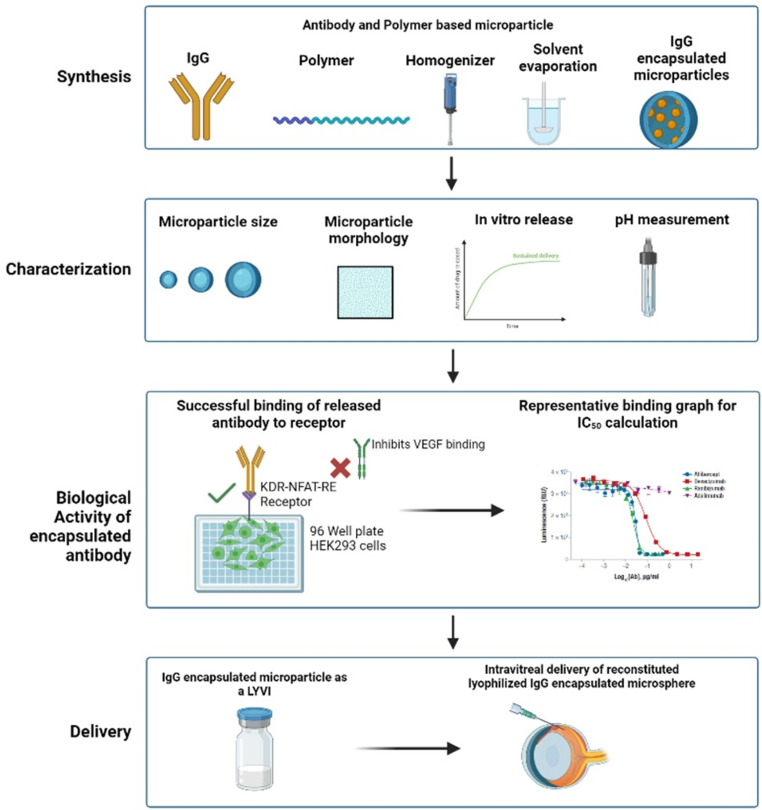

## Introduction

Therapeutic proteins, such as antibodies, are widely used to treat multiple diseases, including the current standard of care for the treatment of posterior eye segment diseases [1]. The current treatment for wet age-related macular degeneration (AMD) includes monthly intravitreal injections of antibody therapeutics targeting the vascular endothelial growth factor (VEGF) such as ranibizumab, aflibercept, and brolucizumab [2]. Following these monthly injections, the patient still needs to be administered with the therapeutic agents once every 12 weeks, or more frequently, to maintain the efficacy of the drug and reduce the progression of the disease. In clinical trials of these anti-VEGF treatments, the overall rate of maintaining visual acuity of patients has been around 90%. However, in real world scenarios, this percentage is closer to 50% as most of the patient population in this disease are elderly and there are significant challenges in maintaining their dosing schedules [3]. Hence, especially for diseases like AMD, a treatment strategy that can deliver the protein drug on a continuous and predictable manner is of critical importance.

In 2021, United States Food and Drug Administration (US-FDA) approved the long-acting port delivery system (PDS) implant, Susvimo^®^, containing anti-VEGF antibody ranibizumab that provided a continuous release of the active biologics for an estimated period of 6 months [4]. The treatment is highly efficacious; however, the implant is placed in the vitreous chamber via surgical intervention and during the LADDER and ARCHWAY clinical trials more adverse events were observed among patients receiving the PDS based therapy than the intravitreal injection of ranibizumab solution. This led the US-FDA to provide a label for PDS treatment with a warning of higher rates of endophthalmitis [5].

With only a single product currently approved by the US-FDA for the sustained delivery of anti-VEGF antibody that could potentially increase patient compliance, we wanted to explore an injectable and bio-erodible drug delivery system strategy that does not require surgery and could pose less safety concerns than the PDS. For this reason, we sought to develop a long-acting anti-VEGF antibody encapsulated in a biodegradable polymeric microsphere to release the biological candidate in a continuous manner over at least 6 months period. In this type of microsphere delivery system, the treatment requires an intravitreal injection instead of a surgical intervention used in an implant, thus reducing risks such as endophthalmitis [6, 7].

Polymeric microspheres are relatively larger particles (as compared to nanoparticles) that range between 1 and 1000 μm in diameter enabling the encapsulation of large biotherapeutics, such as full-length antibodies. Using microspheres as a drug carrier, there is a potential to encapsulate therapeutically active drug and release them in a controlled manner in the eye, thus reducing the frequency of dosing and enhancing patient compliance [8]. Key distinction between the usage of nanoparticles versus microparticles for intravitreal delivery stems from the fact that nanoparticles tend to float in the vitreous chamber, which is not observed with microspheres due to its high density [9]. Considering the small size and an increase in surface area to volume ratio, nanoparticles could deliver the drug faster as compared to microparticles which could not be beneficial for long term drug delivery strategies [10]. Previous studies have demonstrated the encapsulation of bevacizumab using PLGA microspheres and these formulations have been tested in pre-clinical animal models to assess its pharmacodynamic effect and tolerability of the encapsulated microspheres [11]. However, very limited in vivo studies are conducted using PCL as the polymer of choice for encapsulating bevacizumab [12].

The main objective of the present study was to provide an insight into assessing alternative polymers such as PCL, which could provide additional benefits in demonstrating the long-term release behavior of the formulations and keeping the biological activity of the encapsulated drug intact. The process to fabricate microspheres containing hydrophobic therapeutics agents like small molecule drugs has been widely studied and established [13]. However, fabrication of microsphere formulation to encapsulate a large, hydrophilic, protein candidate, such as full-length antibody, has been challenging due to the inherent properties of the molecule (e.g., physical and chemical degradation, charge distribution, propensity to self-associate and aggregate) and the impact of the fabrication process that utilizes organic solvents and high shear rates [14]. Post-process modifications in the amino acids and protein sequence such as isomerization, deamidation and oxidation as well as self-association and aggregation propensity of a protein could result in lower bioactivity of the biotherapeutics thus drastically reducing its in vivo efficacy [15].

We have evaluated the encapsulation of a full-length anti-VEGF antibody, bevacizumab (IgG-1 antibody, molecular weight 149 kDa), using two different polymeric systems: poly (D, L-lactic-co-glycolic acid) (PLGA) and poly(epsilon-caprolactone) (PCL). We assessed various formulation parameters such as concentration of the polymer, inner volume ratio of the primary emulsion, ratio between inner aqueous phase and outer continuous phase, concentration of the surfactant in the continuous phase and the starting protein load to successfully encapsulate a full-length antibody into a microsphere system. We observed superior results by using PCL to encapsulate bevacizumab with respect to its surface morphology, release kinetics and preserving the biological activity of the encapsulated drug. Most of the other characteristics of the microspheres’ formulations such as encapsulation efficiency, drug loading, and size distribution remained similar between the two polymeric systems.

## Materials and methods

### Materials

PLGA also known as Resomer^®^ 503 H (molecular weight 24–39 kDa; lactide: glycolide ratio of 50:50), Poly (vinyl alcohol) (PVA,87–89% hydrolyzed, molecular weight 13–23 kDa, poly(epsilon-caprolactone) (PCL, molecular weight 90 kDa), dichloromethane (> 99.5% purity) and 5 M sodium chloride (NaCl) solution were purchased from Sigma Aldrich. Anti-VEGF antibody, bevacizumab was internally obtained from the Sandoz unit of Novartis, Pharma AG, Schaftenau, Austria. All other chemicals were analytical grade and used without further purification. The fabrication process was carried out using IKA’s T-18 Ultra Turrax homogenizer. The microspheres were lyophilized using the Labconco FreeZone Triad benchtop lyophilizer.

**Fig. 1:**
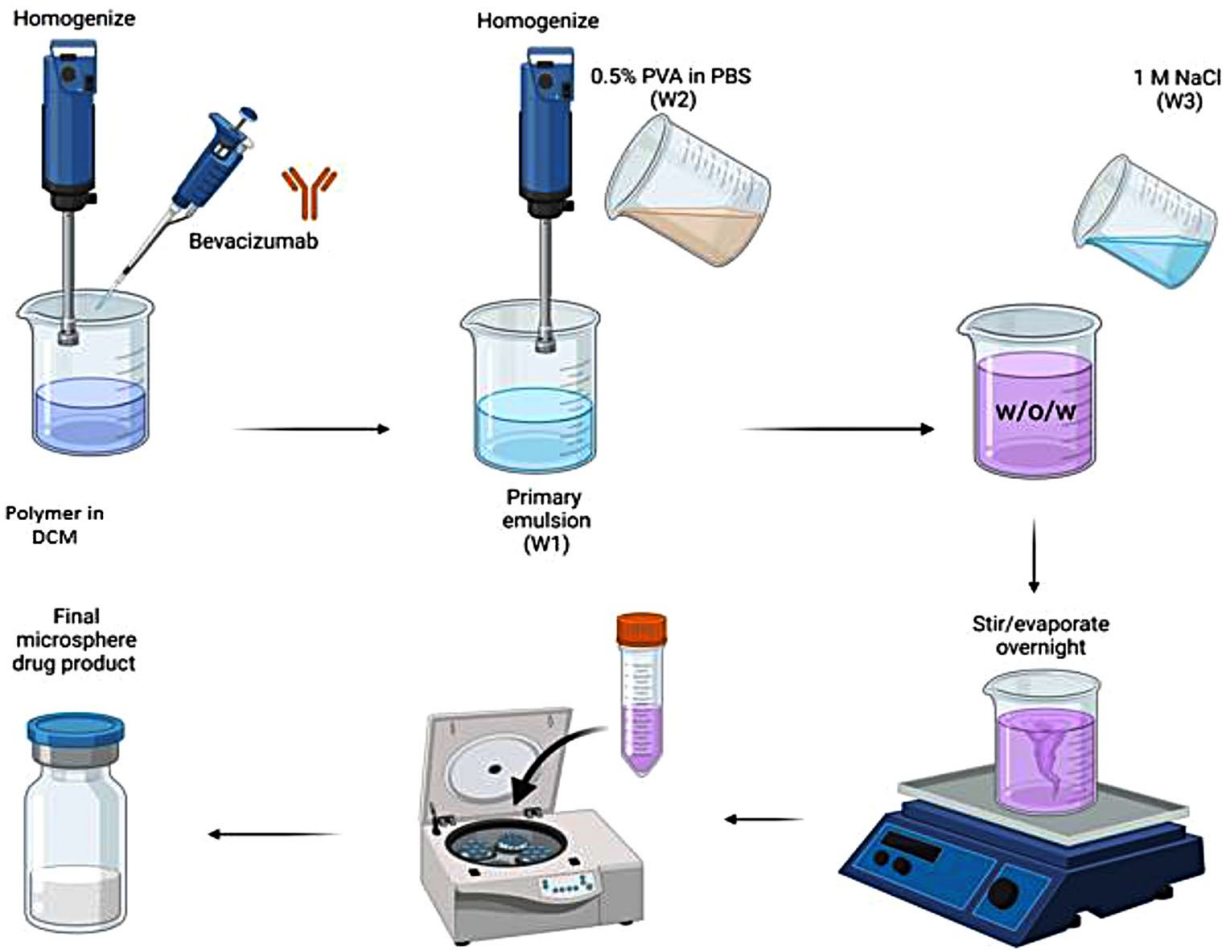
Graphical representation of the antibody encapsulated biodegradable polymeric microsphere fabrication process using the double emulsion method

### Preparation of bevacizumab-encapsulated PLGA and PCL microspheres

After multiple studies conducted (data not shown) to identify preferred conditions for the fabrication process, we devised the following protocol that can be used to fabricate bevacizumab-PLGA microspheres. These microspheres were prepared using the double emulsion-solvent evaporation technique (Fig. 1). The oil phase consisted of 20% (w/v) PLGA, and the inner aqueous phase was the liquid formulation at a starting protein concentration of 25 mg/ml containing sugar and surfactants needed for its stability during the fabrication process. A total of 2 ml of 25 mg/ml (total protein load of 50 mg) liquid formulation of bevacizumab was added to 8 ml of 20% (w/v) PLGA pre-mixed in dichloromethane. The solution was subjected to a sheer stress of 20,000 rpm for 45 s using the Turrax homogenizer.

The resultant mixture formed was the primary emulsion. This primary emulsion was then added to 0.5% (w/v) PVA in PBS to form the secondary emulsion using the Turrax homogenizer at a sheer stress of 20,000 rpm for 45 s. An addition of “extraction phase” containing 1 M NaCl was introduced in the fabrication workflow to maintain an osmotic gradient between the inner aqueous phase and the outer continuous phase. This resultant solution was then stirred overnight inside a chemical hood for complete evaporation of dichloromethane. While the dichloromethane evaporates, the polymer precipitates and hardens resulting in the formulation of microparticles [16]. After overnight evaporation, the solution was centrifuged at 4,000 rpm for 20 min, and the resultant pellet was washed 5–8 times with Milli-Q water. The final washed pellet was then lyophilized to obtain the bevacizumab loaded microspheres powder samples.

The protocol described here for bevacizumab-PCL microspheres was finalized after conducting multiple studies (data not shown) to identify preferred fabrication conditions with the PCL polymers. The two protocols are different as both PLGA and PCL have distinct differences when it comes to absolute viscosity of the polymeric solution in organic solvent, ease of handling the polymer, and its hydrophobic-hydrophilic balance. Thes factors have been demonstrated to contribute to the fabrication process as well as the overall characteristics of the microspheres [17].

Bevacizumab-loaded PCL microspheres were also prepared using the double emulsion-solvent evaporation technique. The oil phase consisted of 3% (w/v) of PCL, and the inner aqueous phase was the liquid formulation at 55 mg/ml of bevacizumab containing sugar and surfactants needed for its long-term stability. Briefly 2 ml of 55 mg/ml of bevacizumab (total protein load of 100 mg) was added to 8 ml of 3% (w/v) of PCL pre-mixed in dichloromethane. This solution was subjected to a sheer stress of 20,000 rpm for 45 s using the Turrax homogenizer. The resultant solution is the primary emulsion. This primary emulsion was then added to 0.5% (w/v) PVA in PBS to form the secondary emulsion using the Turrax homogenizer at a sheer stress of 20,000 rpm for 45 s. An addition of “extraction phase” containing 1 M NaCl was introduced in the fabrication workflow to maintain an osmotic gradient between the inner aqueous phase and the outer continuous phase. This resultant solution was then stirred overnight inside a chemical hood for complete evaporation of dichloromethane. While the dichloromethane evaporates, the polymer precipitates resulting in the formulation of microspheres [16]. After overnight evaporation, the solution is centrifuged at 4,000 rpm for 20 min, and the resultant pellet is washed 5–8 times with Milli-Q water. The final washed pellet is then lyophilized to obtain the final bevacizumab-loaded microspheres.

Based on the above fabrication methods, we obtained the following three formulations:

#### Formulation 1

Bevacizumab-loaded PLGA microspheres prepared **without** the presence of 1 M NaCl in the external phase.

#### Formulation 2

Bevacizumab-loaded PLGA microspheres prepared **with** 1 M NaCl in the external phase.

#### Formulation 3

Bevacizumab-loaded PCL microspheres prepared **with** the presence of 1 M NaCl in the external phase.

### Particle size analysis

Malvern’s Mastersizer 3000 + was used to assess the particle size of the microsphere formulation. The measurement was performed using a wet dispersion technique where 200 µl of the microsphere suspension (before freeze-drying process) was added into a dispersion unit called Hydro SM chamber containing the dispersant, water in this case. These dispersion units are designed to circulate the sample within the Mastersizer measurement cell. Typically, wet dispersions are performed when the sample of interest is also fabricated/available as a liquid [18].

### Scanning electron microscopy (SEM) analysis of the microsphere formulation

SEM was used to assess the surface morphology of bevacizumab-encapsulated PLGA and PCL microspheres. Freeze dried protein-PLGA microspheres were utilized for this experiment. Wet samples tend to readily outgas under vacuum, causing significant damage to the samples as well as to the instrument [19]. Hence, utmost care was taken to completely freeze dry protein loaded PLGA microspheres. The samples were first fixed on a brass stub using double sided carbon tape and all loose particles from the samples were briefly removed by using compressed air. Since polymers are solubilized in organic solvents, they tend to carry charge even at low magnifications and accelerating voltages. Hence, a light gold coating can eliminate most charging effects and optimize resolution, which was performed for all formulations prior to placing the sample loaded stub inside Phenom Pro [19]. This sputter coating of the particles was needed to improve the imaging capability as they do not react with polymeric based samples and produce highly efficient deposition rates due to their molecular weight. The SEM images were determined using the Phenom ProX instrument, from ThermoFisher Scientific using an electron beam energy between 5 and 15 kV based on the type of surface analysis.

### Bevacizumab encapsulation efficiency and drug loading

The percentage encapsulation efficiency and percentage by weight drug load of the microspheres was determined using the direct method via bicinchoninic acid/Bradford (BCA) assay. A solution of 0.1 N NaOH with 5% (w/v) of sodium dodecyl sulfate (SDS) was added to 20 mg of freeze-dried microsphere powder. The resultant solution was left to solubilize (clear appearance) overnight at room temperature. 0.1 N NaOH accelerates the degradation of hydrophobic polymers such as PLGA and PCL, thereby releasing the encapsulated protein which further solubilizes in 5% (w/v) SDS [14]. After overnight incubation, the concentration of the protein was determined by adding 25 µl of the solubilized solution to 200 µl of the Pierce BCA working reagent mix followed by 30 min incubation at 40ºC. The solution absorbance was measured at a wavelength of 562 nm using the Spectramax^®^ plate reader. To obtain the protein concentration in the unknown sample solution, a standard curve of bevacizumab in a concentration range of 1.25 mg/ml − 0.05 mg/ml was generated, and the encapsulated bevacizumab drug concentration is determined using its standard curve. This was referred to as the direct method to calculate percent encapsulation of bevacizumab. The percentage encapsulation efficiency (EE%) and percent drug load (DL%) is calculated by the following formulae:

$$\begin{aligned}&\text{EE}\%\cr&\quad={{{\rm{Amount }}\,{\rm{of }}\,{\rm{protein }}\,{\rm{in}}\,{\rm{ the}}\,{\rm{ encapsulation}}\,{\rm{ buffer}}\left( {{\rm{mg}}} \right){\rm{ }}} \over {{\rm{Initial }}\,{\rm{amount }}\,{\rm{of }}\,{\rm{drug}}\,{\rm{ loaded }}\left( {{\rm{mg}}} \right)}}\,\times\,{\rm{ 100}}\end{aligned}$$

$$\begin{aligned}&{\rm{DL}}\% \cr&\quad= {{{\rm{Amount }}\,{\rm{of}}\,{\rm{ protein}}\,{\rm{ in}}\,{\rm{ the}}\,{\rm{ microsphere}}\left( {{\rm{mg}}} \right)} \over {{\rm{Total}}\,{\rm{ amount }}\,{\rm{of}}\,{\rm{ microsphere}}\,{\rm{ used}}\,{\rm{ for}}\,{\rm{ testing}}}}\,\times{\rm{ 100}}\end{aligned}$$

### In vitro bevacizumab release from polymeric microspheres

An in vitro release study for up to 3 months was performed to study the release kinetics of bevacizumab from PLGA and PCL matrix. Briefly, 20 mg of each microsphere formulation was added to 1 ml of phosphate buffered saline (PBS) and incubated at 40ºC for a period of 3 months. At each timepoint, 400 µl of the sample was withdrawn and fresh 400 µl of PBS was added into the microspheres and the study was continued further. For analysis the samples were centrifuged at 8,000 rpm for 20 min, and the supernatant was used to measure the protein concentration via BCA assay.

### Solution pH measurements

All samples from the in vitro release study were measured for pH using a pH probe meter catalog number 8220BNWP from Thermo Scientific. 40 µl of the release sample at each timepoint was measured in triplicates. Data was expressed as the average of three readings.

### Measurement of encapsulated bevacizumab bioactivity

Promega’s VEGF Bioassay was utilized to assess the bioactivity of encapsulated bevacizumab in two different polymeric systems pre and post fabrication processes as well as during in vitro release studies. The bioactivity assay provides a faster and easier method to study angiogenesis as compared to the use of primary human umbilical vein endothelial cells (HUVEC), such as endothelial cell proliferation and differentiation assay. This experiment was performed to confirm the inhibitory response of bevacizumab to VEGF and subsequent inhibition of VEGF binding to KDR/NFAT-RE receptors on HEK293 cells post fabrication process. Once confirmed, we performed the bioassay to understand if the bevacizumab released from PLGA and PCL microspheres exhibited bioactivity after 2 months. Along with microsphere formulation, liquid formulation of bevacizumab was also used as a control sample to assess its inhibitory activity and compare the IC_50_ values quantified by the two sample sets. Bevacizumab presented as a liquid formulation was thawed in a water bath at room temperature and then diluted to a stock concentration of 18 µg/ml using the formulation buffer. The released samples at day 7 and day 60 were kept frozen until the day of performing the bioactivity assay. The frozen vials were thawed and then centrifuged at 8,000 rpm for 20 min for the polymer pellet to settle at the bottom. The supernatant containing the released protein was used for the measurement. The initial concentrations of the protein solutions were determined using BCA assay. 10 step serial dilutions with the assay buffer were then prepared using the starting concentration using the Promega assay buffer.

On the day of the assay, KDR/NFAT-RE HEK293 cells were removed from − 80^○^C storage and prewarmed assay buffer was added to 0.4 ml of the cell vial. The cell suspension was gently mixed and added to the inner 60 wells of a 96 well plate. All three samples were added to the desired well according to the plate layout in triplicates (three different dilution sets). A recombinant VEGF mixture at a concentration that is 3x the value of its EC_50_ was then added to the desired wells of the 96 well plate. The plate was then incubated for 6 h in a 37^◦^C, 5% CO_2_ humidified incubator. After 6 h, Bio-Glo reagent was added and RLU was read using a standard luminometer. Data expressed as mean ± SD, *n* = 2 and fitted to a 4PL curve using the GraphPad Prism^®^ software.

## Results

### Microsphere size and morphology

**Fig. 2:**
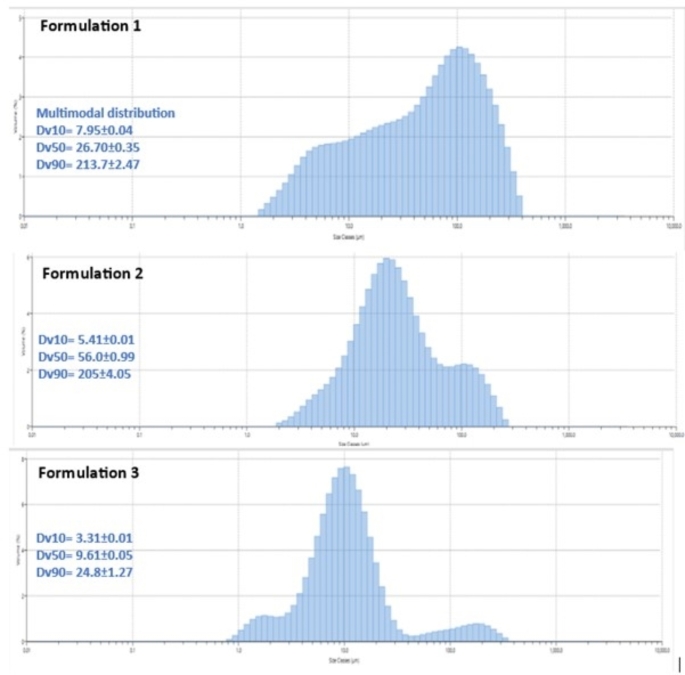
The particle size distribution of bevacizumab-encapsulated PLGA microspheres in Formulation 1, Formulation 2 and Formulation 3. The data is as mean ± SD (*n* = 2)

**Fig. 3:**
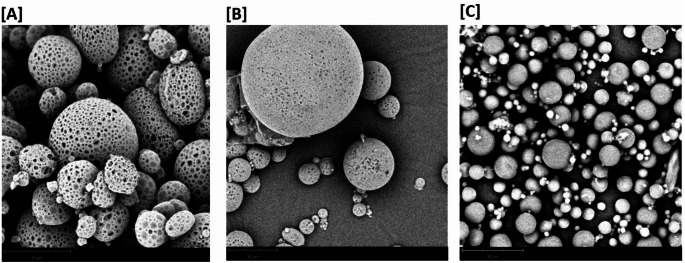
Scanning electron microscopy analysis of the surface morphology of bevacizumab-loaded polymeric microspheres. The figure shows a heterogenous distribution of particles as also measured via size analysis in Fig. 2. (**A**) Formulation 1, (**B**) Formulation 2, and (**C**) Formulation 3. Scale bar = 30 μm

The results in Fig. 2 show the size of bevacizumab microspheres in two different biodegradable polymeric matrices, PLGA and PCL. A heterogenous size distribution was observed in both matrices irrespective of the polymer used. The size distribution remained comparable with the addition of 1 M NaCl in the extraction phase which was incorporated to reduce surface porosity as shown in Fig. 3 (A) and (B). For microspheres fabricated using PLGA, we observed higher size distribution of DV_90_ in the size range of > 100 μm. However, with PCL polymer, the size range remained < 50 μm for all the size population. Surface morphology assessment using SEM demonstrated the positive effect of addition of 1 M NaCl Fig. 3(A) and (B). Without the extraction phase, porous microspheres were observed, which could potentially result in premature burst release of the drug. As compared to bevacizumab microspheres fabricated using PLGA, a much smoother, non-porous surface was observed with bevacizumab microspheres fabricated using PCL Fig. 3(C).

### Bevacizumab encapsulation efficiency and loading

**Fig. 4:**
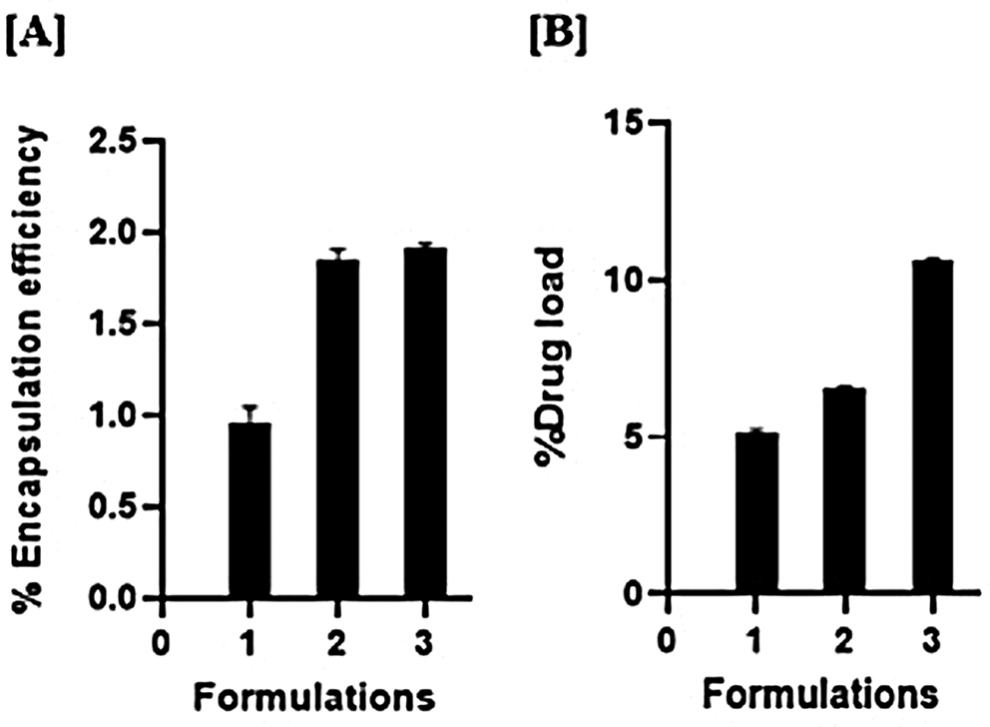
(**A**) Bevacizumab percent encapsulation efficiency and (**B**) percent drug loading in PLGA and PCL microspheres. The samples represent Formulation 1, Formulation 2 and Formulation 3. Data is expressed as mean ± SD (*n* = 2)

Encapsµlation efficiency and drug loading capacity of microspheric formµlations were calcµlated based on a direct method as described in the “In vitro bevacizumab release from polymeric microspheres” section. The data in Fig. 4 shows the percent encapsulation efficiency and percent drug loading of bevacizumab in PLGA microspheres with NaCl (Formulation 1) and without NaCl/extraction phase (Formulation 2) and PCL microspheres (Formulation 3).

The amount of drug encapsulated showed a significant difference between the microspheres generated without the extraction phase and the microspheres generated either with the extraction phase or with PCL. While fabricating microspheres with PLGA and PCL we started with different amounts of bevacizumab as explained in the “Materials and method” section. With PLGA microspheres, our starting load was 50 mg of the total protein whereas with PCL microspheres, our starting load was 100 mg. The higher concentration of PLGA (20% (w/v)) combining with higher starting protein load resµlts in a more viscous solution which was challenging to work with. In contrast, by using lower concentration of PCL (3% (w/v)), we had an opportunity to increase the starting protein load in PCL fabricated microspheres. With this strategy, we were able to achieve better encapsulation and drug loading of the microspheric formµlations. While it is true that much of the literature reports high encapsulation efficiency with this method, it is typically with small size molecules, proteins and peptides and alternative polymers to PLGA. In contrast, our research focuses on the encapsulation of larger antibodies, which presents unique challenges. While there is literature evidence showing the encapsulation efficiency with bevacizumab, we discovered that alternative techniques, such as the S/O/W (solid-in-oil-in-water) method or other technologies utilized in the double emulsion fabrication process, may offer potential benefits in enhancing encapsulation efficiency for large molecules like antibodies [20].

### In vitro bevacizumab release studies

**Fig. 5:**
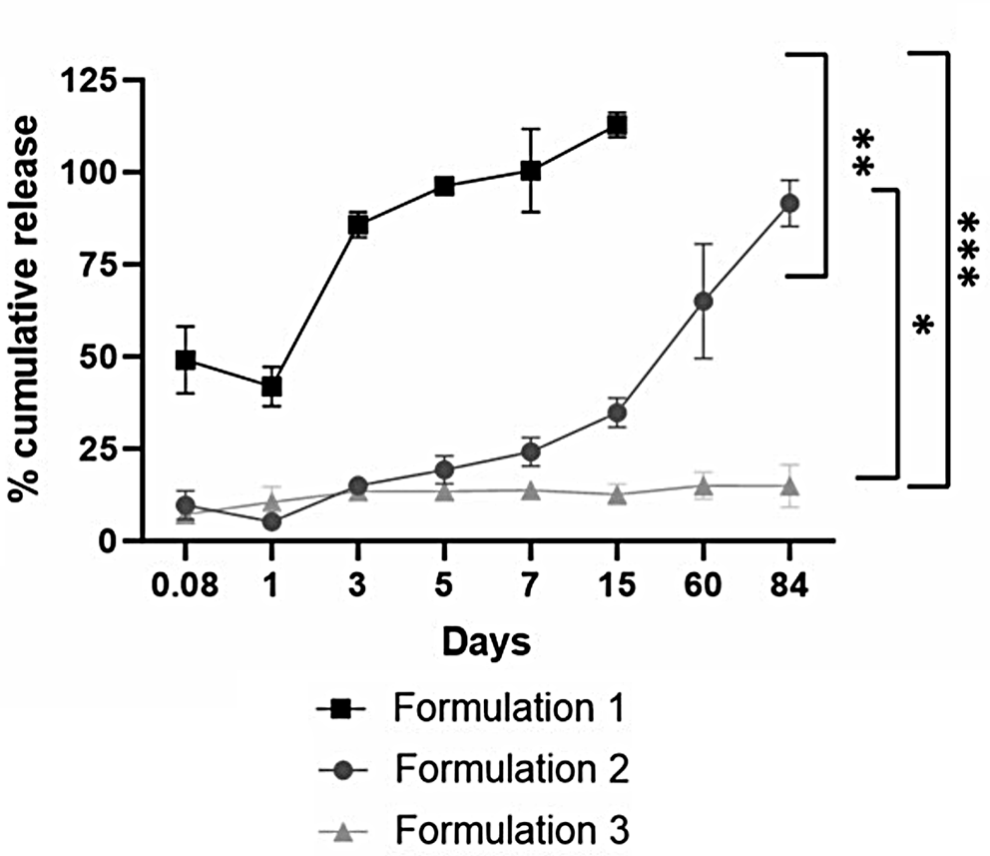
In vitro bevacizumab release in phosphate buffered saline (PBS, pH 7.4) at 40 °C over a period of 3 months from Formulation 1, Formulation 2, and Formulation 3. Data is expressed as mean ± SD (*n* = 2). One-way ANOVA with Tukey’s multiple comparison test *p < 0.05, **p < 0.01, ***p < 0.001

The release of bevacizumab from different microsphere formulations were tested for a period of 90-days. The squared line graph line indicated in Fig. 5 shows the release kinetic of bevacizumab from PLGA microspheres without the extraction phase. We clearly observed a “burst release” of the drug within 2 h of incubation followed by a complete release of the drug within 1 week of its incubation. This could be attributed due to the presence of large pores [19] as observed in the SEM images Fig. 3(A). With the incorporation of an extraction phase of 1 M NaCl, the surface porosity was substantially reduced which in turn impacted the in vitro release of the drug. We observed a 10% release of bevacizumab from the microspheres fabricated using the extraction phase-circle line graph in Fig. 5 as compared to a 50% of drug released from PLGA microspheres without the extraction phase-square line graph in Fig. 5. In contrast, the release of bevacizumab from PCL microspheres was observed to be in a controlled manner with only 10% of the drug being released over a period of 90 days- triangle line graph in Fig. 5. This could be attributed to the slower degradation of PCL as compared to the PLGA used in these studies [18].

### Solution pH changes with polymer degradation

**Fig. 6:**
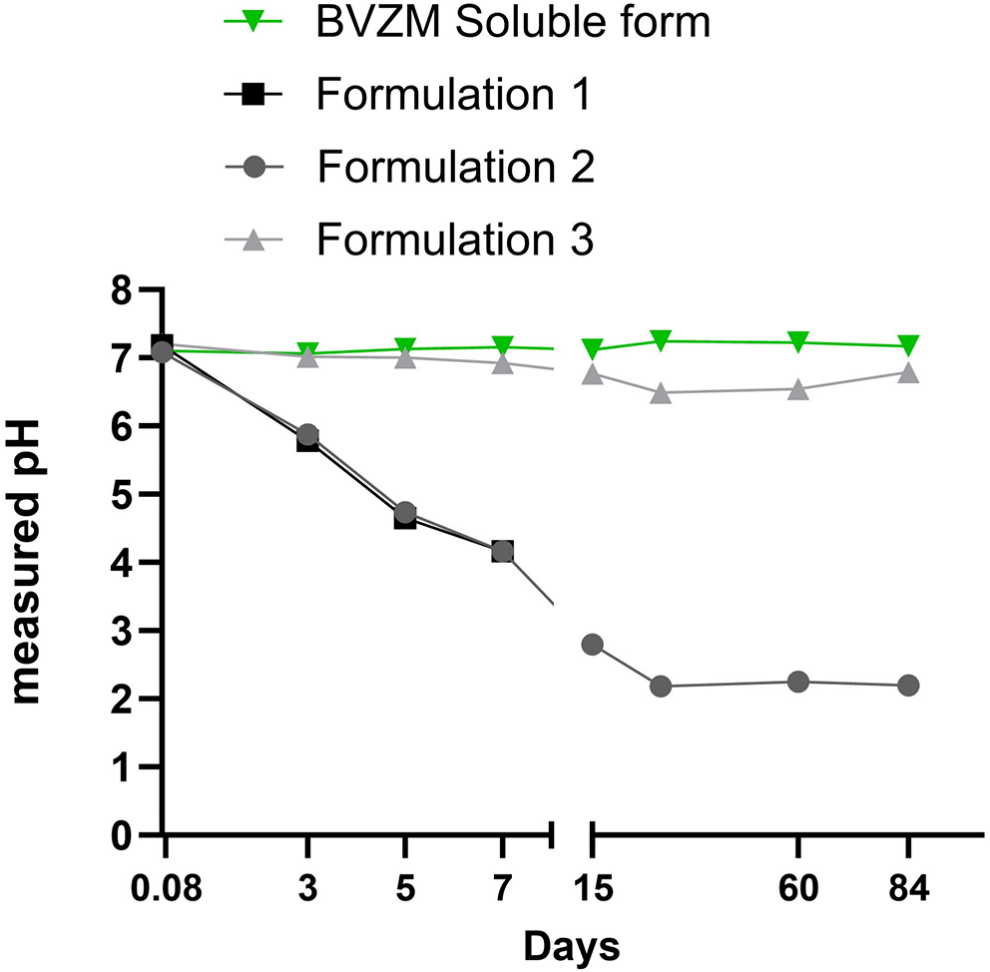
Solution pH changes as a function of time due to polymer degradation during the in *vitro* release experiments in phosphate buffered saline (PBS, starting pH 7.4). The samples represent Formulation 1, Formulation 2 and Formulation 3. Data is expressed as mean ± SD (*n* = 2)

Hydrolytic degradation of PLGA results in the formation of acidic byproducts such as lactic and glycolic acid [21]. This degradation often results into the formation of acidic microenvironment which could impact the encapsulated drug [22]. The data in Fig. 6 shows the sudden drop in pH after 7 days incubation of bevacizumab-PLGA microspheres with and without the extraction phase of 1 M NaCl. The pH values further continued to drop drastically at Day 15 to a pH < 4. When bevacizumab was encapsulated with slow degrading, hydrophobic polyester such as PCL [18], the drop of pH was not as drastic as observed with PLGA microspheres. Due to the increased hydrophobicity of PCL governed by the presence of 5 -CH_2_ moieties in its chemical structure, its degradation rate was lower than PLGA [23] resulting in a much smaller drop in pH. As the pH of the release medium in bevacizumab-PCL microspheres remains close to a neutral pH, the interaction of the encapsulated protein with the acidic degradation byproducts should also remain minimal thus retaining its biological activity. The results in Fig. 6 show a comparison of pH of the release medium measured between two polymeric microspheres (PLGA and PCL) and the soluble formulation of bevacizumab. The soluble bevacizumab liquid formulation shows a stable pH measurement (around 7.4) throughout the study period of 90 days.

### Encapsulated bevacizumab bioactivity analysis

**Fig. 7:**
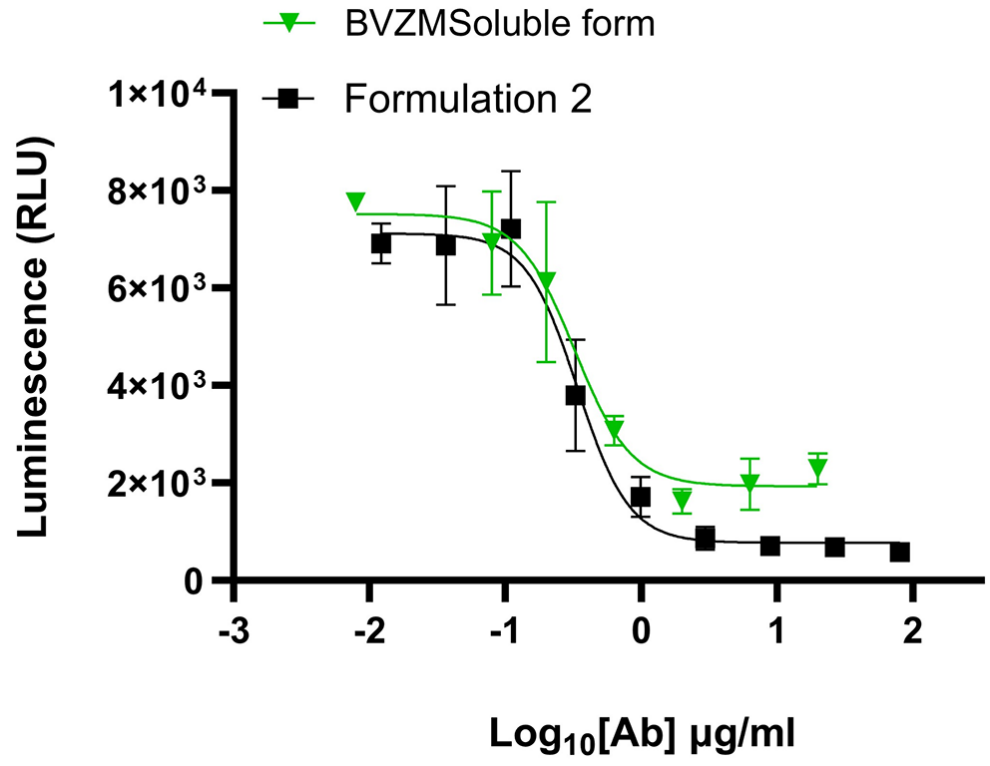
The bioactivity analysis of bevacizumab released from the drug-encapsulated PLGA microspheres made with 1.0 M NaCl in the external phase (Formulation 2) immediately after fabricating the microspheres and compared to the soluble formulation (BVZM-Soluble form) using the Promega VEGF bioassay

Using the in vitro Promega VEGF bioassay, we assessed the inhibitory activity of bevacizumab (i) before and after the W/O/W fabrication process and (ii) released bevacizumab from PLGA and PCL microspheres over 60 days after in vitro incubation. Figure 7 demonstrates that bevacizumab encapsulated using the W/O/W fabrication technology successfully retains its potency by inhibiting VEGF to a similar concentration as observed with the soluble formulation of bevacizumab. The measured IC_50_ values for bevacizumab extracted from microsphere formulations are comparable to the soluble formulation of bevacizumab. The results suggest that the fabrication process for making antibody-loaded PLGA microspheres by itself did not hamper the biological activity of the encapsulated bevacizumab.

**Fig. 8:**
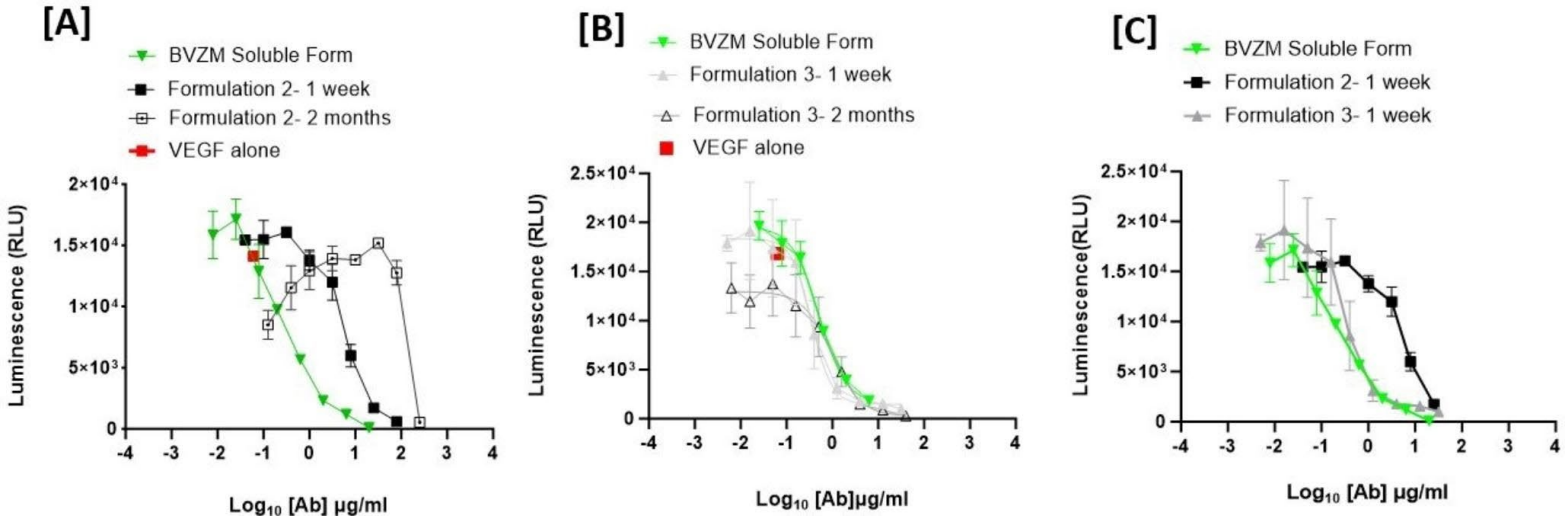
Changes in the bioactivity of encapsulated bevacizumab as a function of time following the release from the polymeric microspheres after 1 week and 2 months. (**A**) The bioactivity analyses from bevacizumab-encapsulated PLGA microspheres made with 1.0 M NaCl in the external phase (Formulation 2). (**B**) The bioactivity analyses from bevacizumab-encapsulated PCL microspheres made without 1.0 M NaCl in the external phase (Formulation 3). The antibody in aqueous buffer (BVZM Soluble form) was used as a control

Once we confirmed the inhibitory activity of bevacizumab to VEGF after the fabrication process, we continued to monitor its potency during our release studies. After 1 week and 2 months of bevacizumab release from PLGA and PCL microsphere samples, the solutions were tested in the VEGF bioactivity assay. The results in Fig. 8(A) show reduced potency of bevacizumab released from PLGA polymer matrix post 1 week and after 2 months incubation as compared to its soluble formulation. We believe this could be due to the drastic drop in pH in Fig. 6 rendering the loss of activity of bevacizumab potentially due to post translational modification or unstable aggregate formation that could happen at low, acidic pH [24]. However, when we performed a similar bioactivity check on the released bevacizumab after 1week and 2 months incubation from PCL microspheres as shown in Fig. 8(B), we observed similar IC_50_ of the anti VEGF antibody as compared to its soluble formulation. As the pH of the release medium remained stable throughout the in vitro release study of bevacizumab encapsulated using PCL polymer, we could establish that bevacizumab showed higher purity and maintained its biological activity throughout the release study.

The results in Fig. 8(C) provide a comparison of the dose response curves of bevacizumab released from PLGA and PCL microspheres after 1 week incubation. We clearly observe the traces of bevacizumab from PCL microsphere overlay with the trace from its soluble formulation and a drastic right shift was observed with the bevacizumab released from PLGA microspheres. Table 1 summarizes the measured IC_50_ values of released bevacizumab from two polymeric microsphere formulations as compared to its liquid formulation.

**Table 1:** Encapsulated bevacizumab bioactivity analyses measured by the IC_50_ values following 1 week and 2 months of release from polymeric microspheres. The samples represent bevacizumab-encapsulated PLGA microspheres made with 1.0 M NaCl in the external phase (formulation 2), and bevacizumab-encapsulated PCL microspheres made without 1.0 M NaCl in the external phase (formulation 3). The antibody in aqueous buffer (BVZM Soluble form) was used as a control

Formulation	Calculated IC_50_ (µM) after 1 week release	Calculated IC_50_ (µM) after 2 months release
BVZM Soluble form	0.0021 ± 0.003	Not measured (as it is a solution and not a microsphere formulation)
Formulation 2	0.0412 ± 0.018	Not determined
Formulation 3	0.0023 ± 0.009	0.006 ± 0.05

## Discussion

While the concept of sustained delivery using encapsulated antibodies has been previously addressed in the labs of Kang and others, our approach aims to provide a more comprehensive understanding by utilizing two different FDA-approved polymers. Our primary focus is on studying the impact of antibody encapsulation in PCL polymers within a rodent study, although these findings have not yet been published. The majority of existing research, such as the study by Zhuo et al., centers around the use of PLGA polymers in a microsphere delivery system and its impact on tolerability. In contrast, our research delves into the impact of using PCL polymers in a microsphere system and aims to further understand its tolerability in naïve animals. In this paper, we have evaluated a controlled release formulation for anti-VEGF antibody by encapsulating it into polymeric microspheres that can be easily administered in the vitreous chamber to obtain slow release of the therapeutic agent. To achieve this goal, we assessed two FDA approved polymers such as PLGA and PCL and studied its benefits and challenges to encapsulate an antibody such as bevacizumab.

Bevacizumab is a full-length monoclonal immunoglobulin antibody that inhibits the binding of vascular endothelial growth factor to its receptor. It has been approved by the United stated food and drug administration for the treatment of metastatic colorectal cancer. However, it has been widely used as an off-label drug in VEGF-mediated diseases such as choroidal neovascularization and diabetic retinopathy [25]. Its mechanism of action is derived by indirectly blocking the binding of VEGF to its receptor, which results in inhibition of angiogenesis and reduces vascular permeability [9]. We chose bevacizumab as a model protein to study its encapsulation characteristics into two biodegradable polyesters: PLGA and PCL, and further assess the potency of drug released from the polymeric matrix using an in vitro VEGF bioassay. PLGA is the most used biodegradable polymer to encapsulate small molecules, proteins and peptides [26]. Our research explored the encapsulation of bevacizumab into PLGA microspheres using the W/O/W technique that encompasses the use of high sheer stress, exposure to organic solvents that are needed to solubilize the polymer and the final freeze-drying process to generate a microsphere powder. With our approach we could successfully encapsulate about 1.5% (w/w) of bevacizumab into PLGA microspheres when measured via direct method as described in section “In vitro bevacizumab release from polymeric microspheres”. Using an in vitro VEGF bioassay, we also demonstrated that the inhibitory action of bevacizumab remains uncompromised even after fabricating the microspheres with high sheer stress conditions and in the presence of organic solvents Fig. 7. The protein released from a PLGA microspheres after 2 months storage at 40 °C was measured for its integrity using size exclusion chromatography. The data confirmed the presence of additional impurities in the sample which could be a result of bevacizumab degradation in the acidic microenvironment. However, during the in vitro release studies we observed a “burst effect “of bevacizumab from PLGA microspheres.

This burst release is governed by the release of a large bolus of the drug before it can reach a stable release rate. In majority of cases, the presence of burst release would be detrimental to the overall release kinetics of a long-acting microsphere system [27]. This proves to be ineffective from a therapeutic point of view and might also require additional doses in the dosing regimen to ensure long-acting nature of the polymeric system [27]. The phenomenon of burst effect could be attributed to the porous surface morphology that we observed when bevacizumab was encapsulated into PLGA microspheres using the classical W/O/W emulsion technique Fig. 3. To mitigate this, we explored various fabrication process parameters to reduce the surface porosity with PLGA microspheres. One such strategy was the introduction of a new phase in the process called the “extraction phase”. This extraction phase consisted of 1 M NaCl which improved its surface porosity. In the W/O/W solvent evaporation technique, when PLGA is solubilized in dichloromethane, it performs as a semi-permeable membrane that restricts the flow of solutes from the inner aqueous phase to the outer continuous phase. As the polymer solution has low solubility in water, water molecules that are entrapped in the inner aqueous phase consisting of the antibody solution can still cross into the outer continuous phase consisting of polyvinyl alcohol. Due to this migration, large pores are generated as the water molecules move outwards and the organic solvent starts to evaporate [19]. With the introduction of an extraction phase consisting of higher molarity of a salt solution such as 1 M NaCl, we were able to generate an osmotic pressure between the inner aqueous phase (W1) and the outer continuous phase (W2). Due to this high osmotic pressure in W2, water flows inwards to a region with low osmotic pressure thus generating smaller pores or cavities on the microsphere surface resulting in lower burst release. Our SEM data revealed the presence of smaller pores on the microspheres generated by the fabrication method consisting of the extraction phase [19]. From our studies, we observed that maintaining a high osmotic pressure in the outer phase could potentially reduce the surface porosity.

With a significant control on surface porosity, our data demonstrated a lower burst effect due to the addition of an extraction phase in the PLGA fabrication process. However, the degradation of PLGA resulted in the formation of an acidic microenvironment over a period of 2 months Fig. 6. This is due to the degradation of PLGA to lactic and glycolic acid. This negatively impacted the inhibitory activity of the drug when the samples were analyzed after 2 months of in vitro release in PBS medium Fig. 8. We confirmed that the medium pH had drastically dropped to as low as pH 4.0 within 15 days of release. Our experiments (data not shown) with the addition of bicarbonates such as ammonium sulfate or any other additives did not show any significant improvement in mitigating the pH of the acidic environment. Our approach to mitigate the formation of acidic microenvironment due to the fast degradation of PLGA was to replace PLGA with another FDA approved biodegradable polymer such as PCL). PCL exhibits slow degradation as compared to its other biodegradable polymer counterparts such as PLGA, PLA, PGA [18]. PCL is an aliphatic polyester polymer that degrades by bulk hydrolysis of the ester bonds. However, due to the presence of five hydrophobic -CH2 moieties in its repeating units, the degradation rate of PCL is the slowest as compared to other biodegradable polymers. Complete degradation of PCL could take as long as 2–3 years [23].

Our results suggest a similar encapsulation efficiency of bevacizumab with PCL polymer using the W/O/W technology Fig. 4. The release kinetics of bevacizumab when encapsulated with PCL as compared to PLGA was slower and released about 10% of the encapsulated drug over two months Fig. 5. We also confirmed the biological activity of the released bevacizumab using Promega VEGF in-vitro bioassay and observed a similar IC_50_ value of released bevacizumab as compared to its commercial liquid formulation. This confirmed the inhibitory activity of the drug after the fabrication process and 1 week post release Fig. 8. The drop in pH in Fig. 6 was also comparatively reduced with the PCL encapsulated drug as compared to its PLGA counterpart, which preserved its bioactivity after 2-month release of the therapeutic drug.

## Conclusion

Our results demonstrated that we could successfully encapsulate bevacizumab into two biodegradable polymers such as PLGA and PCL using the classical W/O/W technology. Both showed similar encapsulation efficiency and were studied for release kinetics over a period of 2 months. The in-vitro release rate of PCL polymer was slower as compared to PLGA polymer and released about 10% of the encapsulated drug even after 2 months. The acidic microenvironment observed with PLGA degradation that negatively impacted the inhibitory activity of the drug was mitigated by using the PCL polymer. The in vitro results described in this paper offers a comparability study of two FDA approved biodegradable polymers: PLGA and PCL and superior characteristic of bevacizumab encapsulated PCL microspheres. Our next scientific question and the direction we are planning to take is to address the tolerability, drug exposure, and syringeability of the formulation containing this heterogeneous population of particles. The literature suggests using a 2–30-gauge needle size for the injectability of these microspheres [28, 29]. Our goal is to explore how these particles behave regarding syringeability through these needle gauges, as well as their tolerability and drug exposure in in vivo models. With this early understanding of PCL microsphere drug delivery system used for encapsulating a full-length antibody such as bevacizumab, we could potentially develop a long-acting therapy for AMD patients.

## Data Availability

Data and materials described in this manuscript will be available upon request.
